# Prognostic value of tricuspid valve regurgitation in patients with pulmonary arterial hypertension and CTEPH: A longitudinal study

**DOI:** 10.1016/j.ijcha.2024.101342

**Published:** 2024-02-17

**Authors:** Kevin M. Veen, Thomas Koudstaal, Paul M. Hendriks, Johanna JM Takkenberg, Karin A. Boomars, Annemien E. van den Bosch

**Affiliations:** a Department of Cardiothoracic Surgery, Erasmus Medical Centre, Rotterdam, the Netherlands; b Department of Pulmonary medicine, Erasmus Medical Centre, Rotterdam, the Netherlands; c Department of Cardiology, Erasmus Medical Centre, Rotterdam, the Netherlands; d ERN-GUARD-Heart: European Reference Network for Rare and Low Prevalence Complex Diseases of the Heart, the Netherlands

**Keywords:** Pulmonary arterial hypertension, Chronic thromboembolic pulmonary hypertension, Tricuspid valve regurgitation, Prognosis

## Abstract

**Aims:**

The prognostic value of functional tricuspid valve regurgitation (TR) in patients with pulmonary arterial hypertension and chronic thromboembolic pulmonary hypertension (CTEPH) remains undetermined. This study primarily aims to quantify the prognostic role of TR in relation to right ventricle (RV) dysfunction on clinical outcomes and secondarily the evolution of TR and RV dysfunction over time.

**Methods:**

Adult PAH or CTEPH patients diagnosed by right heart catheterization were included. Exclusion criteria were prevalent patients and age < 18 years.

The primary endpoint was a composite of death or lung transplantation. Longitudinal evolution of TR and RV dysfunction were modelled with generalized mixed-effect models, which were inserted in a cox model under the joint-modelling framework in order to investigate the association of TR and RV dysfunction with the endpoint.

**Results:**

We included 76 PAH and 44 CTEPH patients (median age:59, females:62 %), with a mean follow-up of 3.2 ± 2.1 years. 31 patients reached the endpoint (2 transplant, 29 mortality). On average the probability of moderate-to-severe TR decreased during follow-up, whereas the probability of moderate-to-severe RV dysfunction remained stable. The cumulative effect of moderate-to-severe TR (HR_per day_ 1.01 95 %CI[1.00–1.01],P < 0.001) and moderate-to-severe RV dysfunction (HR_per day_: 1.01 95 %CI[1.00–1.01],P < 0.001) was associated with the endpoint in univariable joint-models. In a multivariable joint-model with both the evolutions of TR and RV dysfunction only TR remained significant (HR _per day_: 1.01 95 %CI[1.00–1.01],P < 0.001).

**Conclusion:**

Persistent moderate-to-severe tricuspid valve regurgitation during follow-up predicts adverse outcomes and might be a better predictor of lung transplantation and mortality compared to right ventricle dysfunction.

## 1 Introduction

Pulmonary hypertension (PH) is a progressive disease characterized by increased pulmonary vascular resistance leading to elevated pulmonary arterial pressure and increased pulmonary vascular resistance which ultimately leads to right ventricular (RV) failure and premature death [1]. PH can be idiopathic, heritable or associated with multiple other clinical conditions. However, the etiology of pH is still incompletely understood; multiple factors are involved in the pathogenesis of PH, including genetic predisposition, thromboembolic, fibrotic and inflammatory mediators [2], [3], [4], [5]. PH is a heterogeneous disease, subdivided into five subgroups according to the WHO ERS/ESC classification [6]. Currently, even with PH-specific treatment strategies, survival remains poor with a mean 5-year survival of ∼ 60 % for pulmonary arterial hypertension (PAH) [7], [8] and chronic thromboembolic pulmonary hypertension (CTEPH) patients [8], [9], [10]. Because of this poor survival, there is increased need for better prediction of outcome in these patients.

RV dysfunction has been repeatedly shown to be associated with impaired long term outcomes and mortality in patients with PH [11], [12], [13] Nevertheless, the role of tricuspid valve regurgitation (TR) in this interplay remained underexposed. Functional TR may add additional stress to the right ventricle due to volume overload on top of the pressure overload the right ventricle is experiencing. This might further impair right ventricular function and therefore prognosis. In this paper we aim to quantify the prognostic value of TR in patients with pulmonary arterial hypertension. The secondary aim is to objectify the longitudinal evolution of TR and right ventricular function using novel methodology that accounts for multiple repeated measures, adjusted for survival bias.

## 2 Methods

### 2.1 Patients

This prospective observational cohort study was conducted between May 2012 and June 2020. PH patients > 18 years old with a mean pulmonary arterial pressure (mPAP) ≥ 25 mmHg, a wedge pressure ≤ 15 mmHg and a pulmonary vascular resistance ≥ 3WU measured by right heart catheterization were included in the study at diagnosis. PAH and CTEPH patients were diagnosed according to the ERS/ECSC guidelines [6]. Patients were subdivided according to the World Health Organization (WHO) classification [6], [14].

Similar to prior work from our group [15], exclusion criteria were incomplete diagnostic work-up and therefore no confirmed PH diagnosis, not treatment-naïve, age < 18 years, or not capable of understanding or signing informed consent. The study protocol was approved by the medical ethical committee (MEC-2011–392). A written informed consent was provided by all patients. This study was performed conform the principles outlined in the Declaration of Helsinki.

### 2.2 Data collection

Clinical data were collected during the inpatient screening visit for analysis of PH [15]. All patients underwent physical examination by a cardiologist and a pulmonary physician, 6-minute walking test, spirometry, VQ scan, chest computed tomography scan, ultrasound of the abdomen, 12-lead electrocardiography (ECG), transthoracic echocardiography, venous blood sampling and right heart catheterization. Patient characteristics and vital signs were collected, including age, sex, height, weight, systemic blood pressure, heart rate and peripheral oxygen saturation. The New York Heart Association (NYHA) functional class was used to grade the severity of functional limitations. During right heart catheterization, a Swan-Ganz catheter was inserted in the internal jugular vein. A standardized protocol for the work-up of pH was used to obtain hemodynamic measurements and thermodilution or Fick’s principle was used to measure cardiac output [6]. If the obtained capillary wedge pressure was ambiguous, a fluid challenge was performed to distinguish pre-capillary PH from PH due to left heart disease. Data were collected and stored in PAHTool (version 4.3.5947.29411, Inovoltus, Santa Maria da Feira, Portugal), an online electronic case report form. Additionally, all echocardiographic measurements were collected longitudinally via automated extraction from the electronic patient records. Cardiac fuction, including tricuspid annular plane systolic excursion (TAPSE) were performed conform guidelines of the American Society of Echocardiography [16]. Valvular regurgitation and stenosis were evaluated according to the European Association of Echocardiography recommendations [17], [18].

### 2.3 Study outcome

The primary survival outcome in this study was a composite endpoint of mortality or lung transplantation. Mortality and lung transplantation were considered a composite endpoint since it was assumed both composites share similar relative risk reductions and underlying biology is comparable. The secondary outcomes were the longitudinal evolutions of tricuspid valve regurgitation (TR) and RV function, corrected for survival bias. TR was dichotomized and was defined as none-to-mild and moderate-to-severe according to the European society for cardiology (ESC) guidelines [19]. Tricuspid systolic annular plane excursion (TAPSE) was used to quantify RV dysfunction according to the ESC guidelines, and was utilized as a continuous variable in all analyses [19]. Additionally, a semi-quantitative measure of RV dysfunction was used and defined as none-mild impairment and moderate-to-severe impairment. RV dysfunction was determined by an experienced cardiac sonographer based on eyeballing and the parameters described by Lang et al. [20] All echocardiograms were assessed by both the sonographer and a cardiologist. The semi-quantitative RV-dysfunction was dichotomized in the analyses.

### 2.4 Statistical analyses

Continuous data was presented as mean ± standard deviation (Gaussian distribution) or median [interquartile range (IQR)] (nonGaussian distribution). Categorical data was presented as frequencies (percentage).

Linear and logistic mixed-effect models were used to assess evolution of longitudinal biomarkers (TR, TAPSE, RV dysfunction) over time [21]. Random intercepts for patients were incorporated to account for correlations between repeated measurements. Random slopes were added if these improved the models based on Bayesian information criteria. Splines were used to allow for non-linear trajectories over time. Baseline measurement was incorporated as outcome at time point zero.

**Fig. 1:**
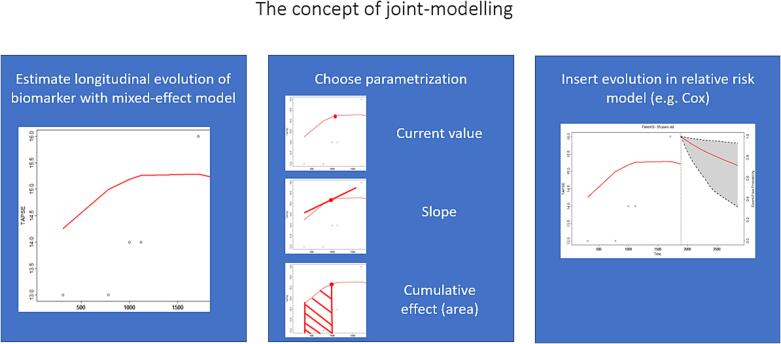
The concept of joint-modelling.

A joint model under the Bayesian estimation approach was developed to investigate determinants of the composite endpoint. More specifically, the mixed-effects models and a relative risk model for the hazard of the endpoint (e.g. Cox model) were jointly modelled using shared-random effects [21]. The subject-specific estimated longitudinal profiles were included in the relative risk model as predictors. Joint modelling has several benefits, such as the appropriate inclusion of endogenous covariates in relative risk models, reduced bias and increased efficiency, while it can be used to derive dynamic predictions, which can be presented as dynamic plots (Fig. 1). At time point *t* one can investigate the effect of the current value of the biomarker (e.g. TR and TAPSE)*,* the effect of the slope of biomarker (at which speed the biomarker is changing at time point *t*) and the cumulative effect of the biomarker, also called parametrizations (Fig. 1). To investigate if moderate-to-severe TR modified the effect of RV dysfunction (a synergistic effect), a joint model including the interaction term between the longitudinal evolution of TR and TAPSE was developed. To communicate the outcomes of joint models effect plots of simulated patients are presented.

Baseline data was missing less than 2 % in all variables and missing mechanism of the data was considered completely at random. Therefore complete-case analysis was used.

Freedom of the composite endpoint was calculated and visualized with the Kaplan-Meier method. Univariable Cox regression was performed to explore associations between the composite endpoint and baseline variables.

A p-value < 0.05 will be considered statistically significant. Statistical analyses were done in R (R core team 2017, Vienna, Austria) with the use of statistical packages “GLMMadaptive”, “splines”, “JointAI”, “survival” and “JMbayes”.

## 3 Results

In total, 120 patients were included in this study. On average patients were 59.5 years old. Seventy-six (63 %) included patients were diagnosed with PAH and 44 patients (37 %) with CTEPH. Baseline variables are presented in Table 1. The mean follow-up was 3.2 ± 2.1 years. After inclusion patients were treated according to the ESC/ERS guidelines, including initiation of PH-specific medical therapy and treatment with BPA or PEA in CTEPH patients [6].

**Table 1:** Demographic and patient variables.

	**PAH** **(n = 76)**	**CTEPH** **(n = 44)**	***p* Value**
**Baseline clinical variables**			
Gender, female (%)	55 (72 %)	20 (45 %)	
Age, y	55.2 ± 16.1	61.1 ± 14.7	**0.044**
BMI, kg/m2	28.4 ± 9.1	28.6 ± 5.7	0.336
NYHA class			
1, n (%)	5 (7 %)	6 (14 %)	
2, n (%)	23 (30 %)	17 (39 %)	
3, n (%)	35 (46 %)	20 (45 %)	
4, n (%)	13 (17 %)	1 (2 %)	
Sinus rhythm	70 (92 %)	42 (95 %)	
6MWT, m	368 ± 128	386 ± 136	0.662
NT-pro BNP, pmol/L	302 ± 642	135 ± 204	**0.004**
Underlying disease			
IPAH, n (%)	18 (23 %)		
HPAH, n (%)	5 (7 %)		
CHD-PAH, n (%)	9 (12 %)		
CTD-PAH, n (%)	26 (34 %)		
Drugs and toxins, n (%)	3 (4 %)		
Auto-immune, n (%)	3 (4 %)		
Po-PH, n (%)	9 (12 %)		
PVOD, n (%)	3 (4 %)		

**Baseline right heart variables**			
mPAP, mmHg	47.6 ± 14.5	40.9 ± 12.5	**0.013**
mRAP, mmHg	10.8 ± 5.8	9.4 ± 6.6	0.073
Capillary wedge pressure, mmHg	11.2 ± 6.2	12.0 ± 4.5	0.072
PVR, wood units	7.9 ± 4.5	4.1 ± 2.4	**< 0.0001**
Cardiac index, l/min/m^2^	2.7 ± 0.8	2.7 ± 0.9	0.92

**Intervention received**			
PEA, n (%)		10/44 (23 %)	
BPA, n (%)		12/44 (27 %)	

**PH-Medication**			
At baseline, n (%)	0/76 (0 %)	0/44 (0 %)	

Data given as ‘mean, ±SD’, unless otherwise indicated.

**Abbreviations:** BMI, body mass index; CTEPH, chronic thromboembolic pulmonary hypertension; PAH, pulmonary arterial hypertension; IPAH, idiopathic pulmonary arterial hypertension; HPAH, heritable pulmonary arterial hypertension, CHD, congenital heart disease; CTD, connective tissue disease; Po-PH, porto-pulmonary hypertension; PVOD, pulmonary veno occlusive disease; 6MWT, 6-minute walk test; NT-pro BNP, The N-terminal prohormone of brain natriuretic peptide; mPAP, mean pulmonary arterial pressure; mRAP, mean right atrium pressure; PVR, pulmonary vascular resistance;

### 3.1 Composite endpoint

**Fig. 2:**
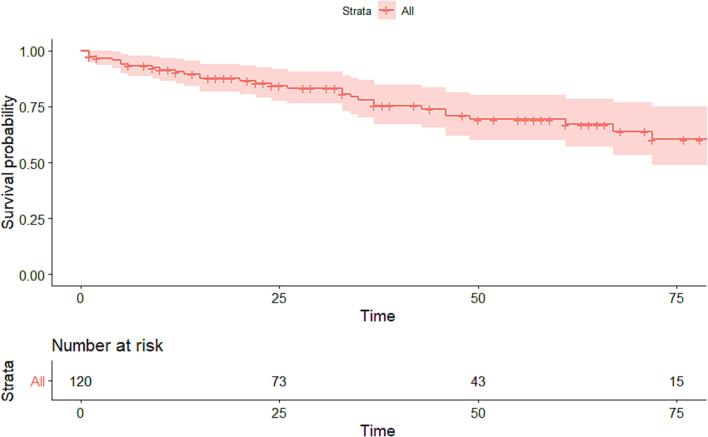
Kaplan Meier curve of mortality or transplantation.

The composite endpoint was reached in 31 patients (lung transplant: 2, mortality: 29). Freedom of the composite endpoint was 69.4 ± 5.0 % at 50 months post-diagnosis (Fig. 2). Older age, NYHA functional class, NT-pro-BNP, mPAP and non-sinus rhythm at baseline were associated with worse outcome based upon univariable Cox regression (Table 2).

**Table 2:** Univariable cox regression of baseline variables potentially influencing time-related LTX/death.

**Variables**	**Hazard ratio (95 % CI)**	**P value**
Age	1.03 (1.00 to 1.06)	0.03
Male sex	0.87 (0.41 to 1.84)	0.71
BMI	1.02 (0.98 to 1.05)	0.36
mPAP	1.03 (1.00 to 1.05)	0.02
mRAP	1.04 (0.99 to 1.10)	0.14
BNP (per 100)	1.07 (1.04 to 1.11)	**<** 0.001
Sinus rhythm	0.34 (0.12 to 0.97)	0.045
NYHA*	2.87 (1.40 to 3.73)	0.001

*As continuous variable.

### 3.2 Longitudinal evolution tricuspid regurgitation

TR was recorded 252 times in 91 patients (mean: 2.7 times, range: 1 – 8). Average longitudinal evolution is presented in Fig. 2B. Probability of TR decreased significantly over time (OR_per year_: 0.57 95 % CI[0.41 to 0.83], P < 0.001).

### 3.3 Longitudinal evolution right ventricle dysfunction

**Fig. 3:**
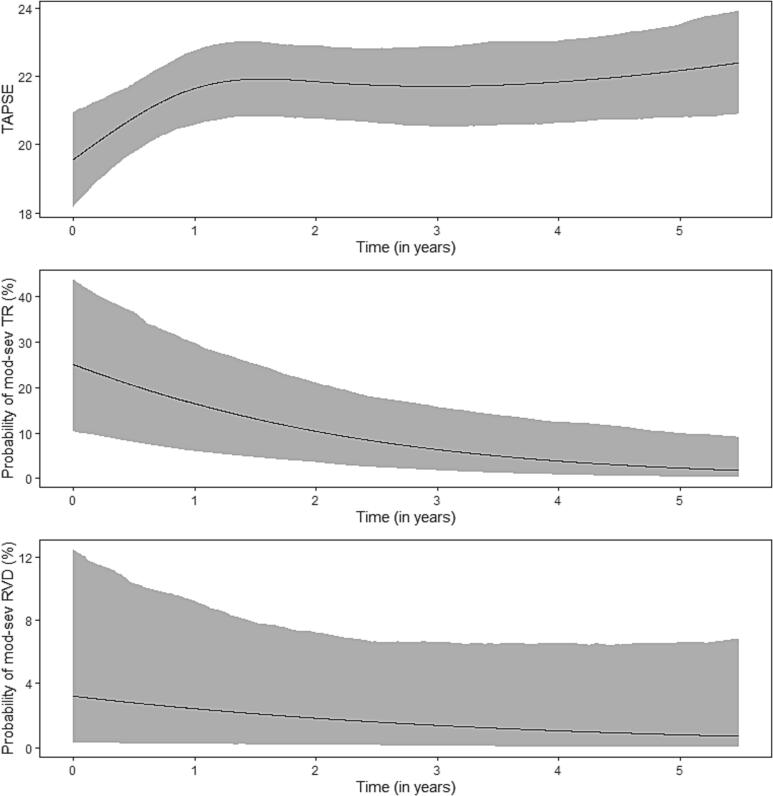
Effectplot of longitudinal evolution is tricuspid annular systolic excursion (TASPE) (A), probability of moderate-to-severe tricuspid regurgitation (B) and probaility of moderate-to-severe right ventricle dysfunction (C).

TAPSE was recorded 275 times in 91 patients (mean 3.0 times, range: 1 – 8) and semi-quantitative right ventricular dysfunction was recorded 242 times (mean 2.7, range 1–8). Average longitudinal evolution of TAPSE and RV dysfunction is presented in Fig. 3A-C, as estimated by the mixed-model part of the joint model. In an average patient, TAPSE increased early after inclusion and stayed relatively stable thereafter. RV dysfunction remained stable over the time period (OR_per year_: 1.00 95 % CI[0.99 to 1.00], P = 0.19). The maximum probability of moderate-to-severe RV dysfunction was 3 % at any timepoint in an average patient.

### 3.4 Evolution right ventricle dysfunction linked to endpoint

TAPSE was not found to be associated with the endpoint in all parametrizations. Unfortunately, the cumulative effect of TAPSE could not be estimated due to convergence issues. The cumulative effect of RV dysfunction was associated with the composite endpoint (Table 3). Other parametrizations were not found to be significantly associated with the outcome.

**Table 3:** Relative risk part of the joint models.

**Variables**	**Hazard ratio (95 % CI)**	**P value**
*Joint model 1*: The relative risk including the cumulative effect parametrization of the longitudinal evolution of right ventricle dysfunction		
Age	1.04 (1.02 to 2.05)	**<** 0.001
Cumulative effect right ventricle dysfunction	1.00 (1.00 to 1.01)	**<** 0.001
*Joint model 2:* The relative risk part including cumulative effect parametrization the longitudinal evolution of tricuspid regurgitation.		
Age	1.02 (0.98 to 1.06)	0.19
Cumulative effect tricuspid regurgitation	1.01 (1.00 to 1.01)	**<** 0.001
*Joint model 3:* The relative risk including both cumulative effect parametrization of the longitudinal evolution of tricuspid regurgitation and right ventricle dysfunction		
Cumulative effect right ventricle dysfunction	1.00 (0.99 to 1.00)	0.83
Cumulative effect tricuspid regurgitation	1.00 (1.00 to 1.01)	**<** 0.001

### 3.5 Evolution tricuspid valve regurgitation linked to endpoint

**Fig. 4:**
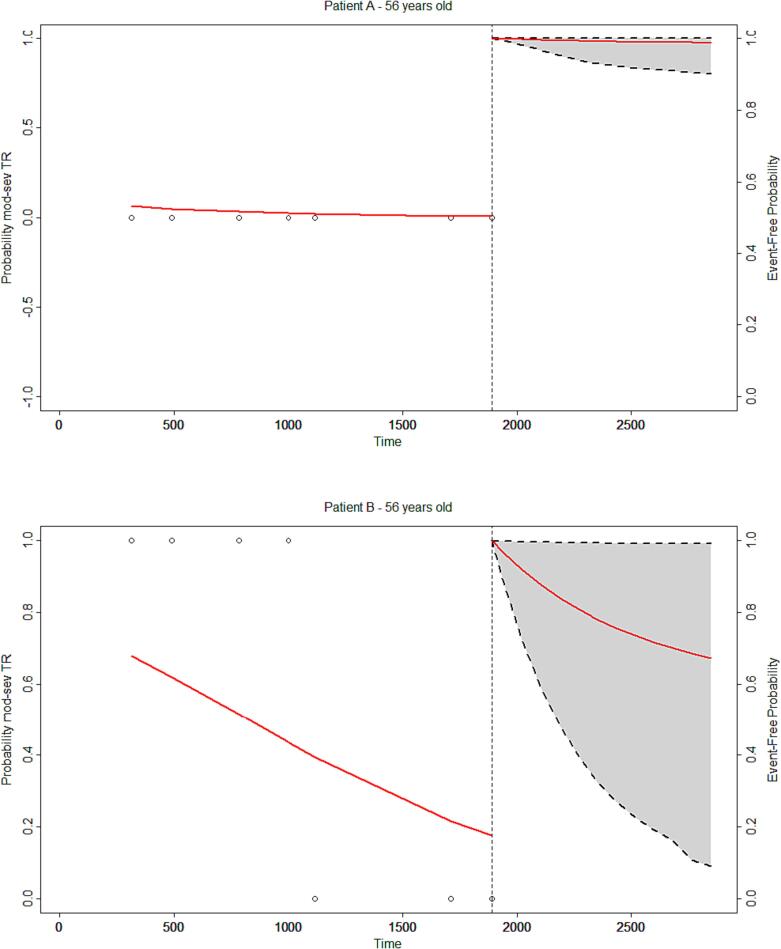
Dynamic survival plot of two patients. Patient A does not develop moderate-to-severe tricuspid regurgitation (A). Patient B starts with moderate to severe tricuspid regurgitation diminishing at apprixmaetley 1100 days after diagnoses and still had impaird survival thereafter.

Moderate-to-severe TR at timepoint *t* was not associated with the composite endpoint. Nevertheless, longstanding TR (i.e. the cumulative effect of TR) was associated with the composite endpoint (Table 3). Fig. 4AB presents to the effect plots of two simulated patients. Patient A did not have moderate-to-severe tricuspid regurgitation during follow-up and patient B had moderate-to-severe tricuspid regurgitation in the first years of treatment. Estimated freedom from the composite endpoint 2.6 years later is 98 % in patient A, whereas this is estimated to be 75 % in patient B. (Fig. 4AB).

### 3.6 Multivariate joint model containing tricuspid regurgitation and right ventricular dysfunction

In a joint model containing both the longitudinal evolution of TR (cumulative effect parametrization) and RV dysfunction (cumulative effect parametrization), only TR remained significant (Table 3). This indicates that longstanding TR is a better predictor than current value right ventricular dysfunction in predicting the composite endpoint.

### 3.7 Synergistic effect TR and right ventricle dysfunction

Simultaneously having TR did not modify the effect deteriorating RV function (HR 0.93 95 % CI [0.63 to 1.51], P = 0.68) (both current value parametrization). In a multivariate mixed-effect model with both TR and TAPSE containing linear random slopes, the random slope of TR and TAPSE were not considerably correlated (R = 0.09), whereas the random slopes of RV dysfunction and TR were considerably correlated (R = 0.32) in a multivariate longitudinal model containing RV dysfunction and TR.

## 4 Discussion

In this paper we explored the prognostic value of tricuspid valve regurgitation (TR) in predicting the composite endpoint of death or lung transplantation in patients with PAH and CTEPH. It was noted that the cumulative effect of TR was associated with the endpoint, whereas the current value was not. This may indicate that in these patients’ treatment for pulmonary hypertension has only limited effect as TR persist and RV remodelling is not or might be less present. Therefore, persistent tricuspid valve regurgitation might be a good marker for adverse outcome.

Not surprisingly, persistent semi-quantitative RV dysfunction was associated with the composite endpoint. Multiple prior studies noted that right ventricular dysfunction is associated with adverse outcome in patients with PAH and CTEPH [11], [12], [13]. However, when RV dysfunction measured by echocardiography and tricuspid valve regurgitation are modelled together in a multivariable joint model with two longitudinal evolutions, only tricuspid valve regurgitation remained a significant predictor of the endpoint. Needless to say, the fact that persistent TR emerged in these models as prognostic predictor does not imply causality. Multiple explanations can underlie these interesting observations related to either statistical features or pathophysiological pathways.

First of all, several compensation methods exist to maintain adequate RV function before end-stage RV failure develops, such as RV hypertrophy and dilatation, which may lead to functional tricuspid valve regurgitation. Hence, functional tricuspid valve regurgitation may precede right ventricular dysfunction. In case of lung transplantation, we tend to transplant patients before irreversible RV function is established. This may result to the fact that prodromes of RV dysfunction (e.g. functional tricuspid valve regurgitation) emerge as significant predictors for the composite endpoints instead of RV dysfunction itself.

Furthermore, functional tricuspid valve regurgitation can mask right ventricular dysfunction, as tricuspid valve regurgitation can function as mode for venting. In fact, to this day, there is still uncertainty whether TR is an independent factor of outcome or rather a surrogate marker of right ventricular disease. In literature, prognosis in PAH is associated with RV function and targeted therapy aims to reduce RV afterload with a concomitant improvement in RV function. Thirdly, in this cohort prevalence of right ventricular dysfunction is low and stable over time. This may indicate that there is not enough variability in RV dysfunction in this cohort to emerge as a significant predictor in statistical models.

Finally, measuring RV dysfunction with 2D echocardiogram is suboptimal and tricuspid valve regurgitation may be measured more reliably compared to TAPSE and eyeballing RV dysfunction. In any case, volumetric data of the right ventricle could help to understand how RV function is changing, positively or negatively and can take into account TR severity as persistent severe TR should be associated with increase in RV end-diastolic volume (prognostically negative signs); reduction in TR should be associated with stability or reduction of RV end diastolic volume (prognostically positive signs).

As aforementioned, residual TR may paradoxically work to off-load the failing RV and systolic function of the right ventricle is overestimated [22]. Nevertheless, a synergistic or antagonistic effect of both RV dysfunction and tricuspid valve regurgitation was not noted, however due to limited sample size only the current value parametrization could be modelled.

In our longitudinal evolution analysis TR decreased over time and right ventricle function increased over time. This might reflect the fact that upon inclusion in the study patients receive PH treatment, decreasing PH pressures and, subsequently, promote RV remodeling.

### 4.1 Strengths and limitations

The major strength of this study is that we were able to account for the possibility of change of either tricuspid valve regurgitation or RV dysfunction over time during follow-up and to account for the dependence between survival status and measurements of tricuspid valve regurgitation and RV dysfunction using the joint modelling framework. Unfortunately, these models require a lot of data to converge and, therefore, we could not perform extensive modelling nor account for many confounders. In this study we combined both PAH and CTEPH, which have a different prognosis. We do however believe that the prognostic value of RV-function does not differ between these diagnostic groups Another limitatation is the fact that we only had 2D echocardiograms available. Data derived form 3D echocardiograms and MRI (including volumetric data) could elucidate the complex intertwined relation of TR and RV dysfunction.

## 5 Conclusion

Persistent tricuspid valve regurgitation during follow up is associated with the composite endpoint of mortality or lung transplantation. Hence, if functional valve tricuspid regurgitation does not decrease under optimal therapy, this may be a marker of an impending adverse outcome. As a prognostic marker, persistent tricuspid valve regurgitation may be a better predictor than RV dysfunction evaluated by 2D echocardiography for the necessity of lung transplantation. This does not imply causality; it may be that persistent TR is a marker for the failing right ventricle and subsequent outcomes.

## Funding

This research project was supported by an unrestricting grant from Actelion Pharmaceuticals.

## CRediT authorship contribution statement

**Kevin M. Veen:** Writing – review & editing, Writing – original draft, Validation, Methodology, Investigation, Formal analysis, Data curation, Conceptualization. **Thomas Koudstaal:** Writing – review & editing, Writing – original draft, Validation, Methodology, Investigation, Data curation, Conceptualization. **Paul M. Hendriks:** Writing – review & editing, Writing – original draft, Validation, Project administration, Methodology, Investigation, Data curation. **Johanna JM Takkenberg:** Writing – review & editing, Supervision, Methodology, Conceptualization. **Karin A. Boomars:** Writing – review & editing, Supervision, Funding acquisition, Conceptualization. **Annemien E. van den Bosch:** Writing – review & editing, Validation, Supervision, Methodology, Funding acquisition, Conceptualization.

## Declaration of competing interest

The authors declare that they have no known competing financial interests or personal relationships that could have appeared to influence the work reported in this paper.
